# Investigating the nutritional status characteristics of terminal cancer patients by the type of cancer

**DOI:** 10.20407/fmj.2024-007

**Published:** 2025-04-17

**Authors:** Norimasa Tsuzuki, Masanobu Usui, Akihiko Futamura, Miyo Murai, Akihiro Ito

**Affiliations:** Department of Surgery and Palliative Medicine, Fujita Health University, School of Medicine, Toyoake, Aichi, Japan

**Keywords:** Cachexia, Nutritional status, Terminal cancer, Gastrointestinal cancer, Gastrointestinal tract cancer

## Abstract

**Objective:**

Patients with terminal cancer experience malnutrition due to cachexia and other problems associated with the disease’s progression. Particularly, patients with gastrointestinal cancers often experience malnutrition because of gastrointestinal symptoms; however, there are few reports evaluating nutritional status based on cancer type up to the prediction of prognosis. In the present study, we examined nutritional evaluation and prognosis based on cancer type.

**Methods:**

In 2019, 234 patients were admitted to Fujita Health University Nanakuri Memorial Hospital and subsequently died before being discharged. Of these patients, 210 were included in the study. Twenty-four patients who were determined to have refractory cachexia on admission were excluded. The 210 patients were divided into two groups, 94 and 116 patients with gastrointestinal cancers and non-gastrointestinal cancers, respectively. Subsequently, data, such as age, sex, presence or absence of metastasis, whether the cancer was initial or recurrent, serum albumin (Alb) and transthyretin (TTR) levels on admission, and survival time were examined. Moreover, for further analysis, the 94 patients with gastrointestinal cancers were classified into 51 and 43 with hepato-biliary-pancreas cancer and gastrointestinal tract cancers, respectively.

**Results:**

Alb and TTR values were significantly lower in patients with gastrointestinal cancer than in patients with non-gastrointestinal cancer (p=0.015 and 0.002, respectively), and Alb values were significantly lower in patients with gastrointestinal tract cancer than in patients with hepato-biliary-pancreas cancer (p=0.049).

**Conclusion:**

Patients with terminal cancer having poor nutritional status exhibit poor prognosis. Particularly, among patients with gastrointestinal tract cancer have exceptionally poor nutritional status. Therefore, providing nutritional management that combines intravenous nutrition with appropriate adjustments to each patient’s gastrointestinal and absorptive condition is important.

## Introduction

Terminal cancer patients have difficulty with oral ingestion and experience malnutrition due to issues, such as functional disability and cancer cachexia, associated with cancer progression. Many patients with terminal cancer exhibit weight loss accompanied by nutritional disorders and are often in a state of cachexia. Cachexia, a metabolic disorder, is characterized by a decrease in skeletal muscle mass accompanied with inflammatory symptoms, affecting patients with chronic debilitating diseases, including cancer, chronic heart failure, chronic respiratory failure, and collagen diseases.^1^ Particularly, when metabolic abnormalities associated with cancer progression are aggravated, it becomes difficult for patients to regain lost weight and muscle mass. Therefore, it is extremely important to prevent the progression of malnutrition. In other words, to treat cancer cachexia, it is necessary to provide nutritional management depending on the stage of cancer. Thus, it is extremely important to accurately grasp the nutritional status of patients with cancer and formulate and implement appropriate treatment plans. However, although it is recognized that there are differences in nutritional status depending on the type of cancer, few articles report on such differences. Patients with gastrointestinal cancer often experience malnutrition because of accompanying specific gastrointestinal symptoms, and there have been few reports evaluating nutritional status based on cancer type up to prediction of the prognosis. In the present study, we examined nutritional evaluation and prognosis based on cancer type on admission.

## Methods

**Figure 1:**
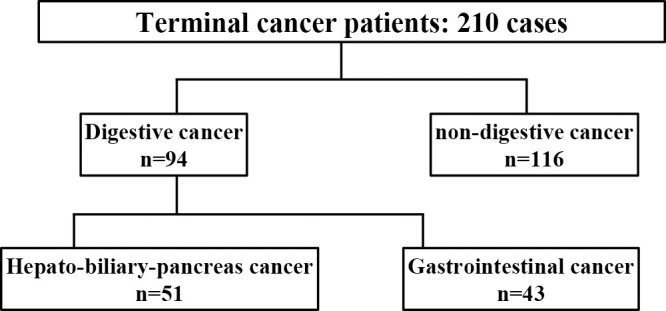
Algorithm of patients with terminal cancer based on type of cancer in Nanakuri Memorial Hospital from January 2019 to December 2019.

This study included 210 patients who were admitted to the Palliative Care Unit of Fujita Health University Nanakuri Memorial Hospital between January to December 2019; these patients either succumbed to the disease or were discharged. We excluded 24 patients who were determined to have refractory cachexia on admission. The 210 patients were divided into two groups, 94 with gastrointestinal and 116 with non-gastrointestinal cancer. Then, age, sex, status of metastases, whether the cancer was initial or recurrent, Alb and TTR values on admission, and survival time were examined. The 94 patients with gastrointestinal cancers were further divided into 51 with hepato-biliary-pancreas cancer and 43 with gastrointestinal tract cancer, and examined similarly. The Alb and TTR levels could not be measured on admission in 2 and 10 patients, respectively. Figure 1 shows the algorithm for the number of patients in each group. To compare the mean values between the two groups we used Mann–Whitney’s U-test, and the Kruskal–Wallis test was used to compare the median values. SPSS.Statistics 27 (Hitachi IBM Corporation) was used for the analysis.

## Results

**Figure 2:**
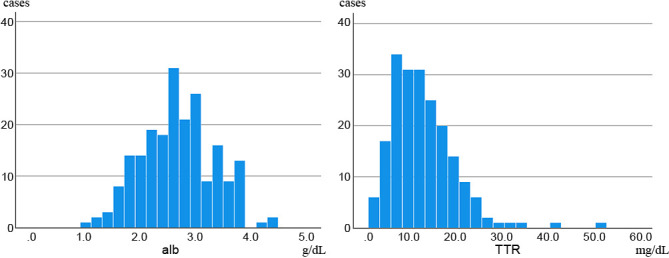
Histogram of serum albumin and transthyretin levels in patients with terminal cancer.

**Figure 3:**
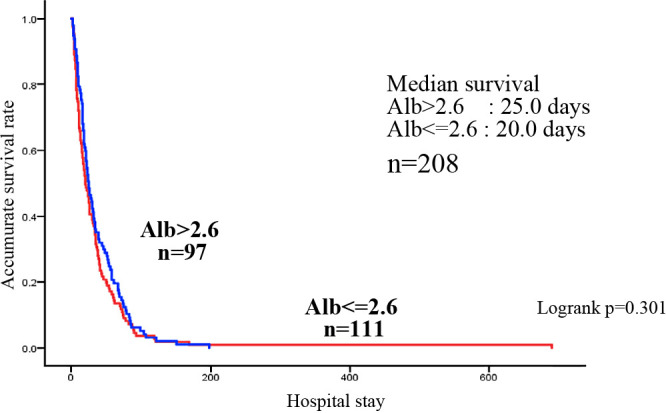
Survival curves of patients with terminal cancer according to albumin level.

**Figure 4:**
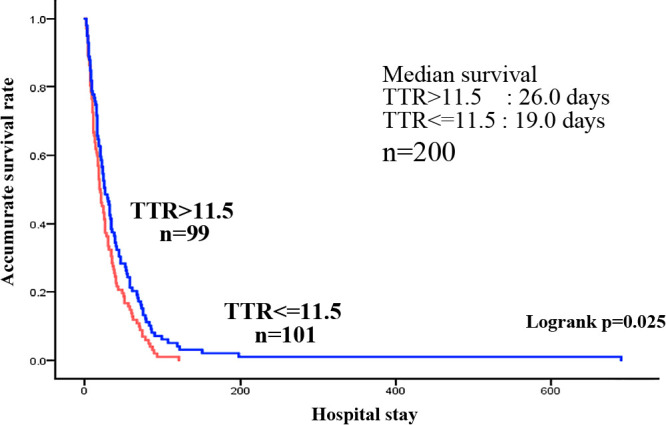
Survival curves of patients with terminal cancer according to transthyretin level.

The study was conducted on 210 patients, excluding 24 patients who were diagnosed with refractory cachexia by a multidisciplinary team on admission and those who died within a few days of admission (1–6 days). Of the 210 patients, 94 had gastrointestinal cancers (4, 15, 24, 34, 6, and 11 cases of esophageal, stomach, colon, pancreatic, hepatic, and biliary tract cancers, each) and 116 had non-gastrointestinal cancers (13, 49, 5, 14, and 16 cases of head and neck, lung, breast, uterine/ovarian, and urological cancers, each, as well as 19 other cancer type) (Table 1). Of the 210 patients, 110 were men and 100 were women. The median age (25%–75%) was 78.0 (69–84) years. Distant metastasis was observed in 136 (64.8%) patients, recurrence in 140 (66.7%), and elevated tumor markers in 131 (62.4%). The median (25%–75%) Alb level at the time of admission was 2.6 (2.2–3.0) g/dL, and the median (25%–75%) TTR level was 11.5 (7.9–16.7) mg/dL, both of which were below the lower normal limit. The median survival time (MST) (25%–75%) was 24.0 (11.0–48.5) days, with more than half of the patients dying within 1 month (Table 1). In the univariate and multivariate analyses performed on factors affecting survival, the univariate analysis showed that age and TTR values tend to contribute to survival, and the multivariate analysis showed age as an independent factor of survival (Table 2). The multivariate analysis revealed that TTR values tended to contribute to survival. Focusing on the relationship between TTR values and survival time, Alb values and TTR values were analyzed for nutritional assessment. In the patients with terminal cancer, blood data shows a high degree of variability, as shown in the histogram (Figure 2). The mean TTR value was 11.9 mg/dL, but the proportion of patients with a value <10 mg/dL was high. Because of the high variability in Alb and TTR values at admission, the median values were used for the study. The median Alb level of all 208 patients was 2.6 g/dL, and the median TTR level of the 200 patients was 11.5 mg/dL. Survival curves showed that the MST was 25.0 days for the group with a median Alb >2.6 g/dL (high Alb group) and 20.0 days for the group with an Alb <2.6 g/dL. Although the MST was slightly longer in the high Alb group, based on the MST, there was no significant difference between the two groups (Figure 3). Conversely, the group with TTR values higher than the median of 11.5 mg/dL (high TTR group) had an MST of 26.0 days, whereas the group with TTR values <11.5 mg/dL (low TTR group) had an MST of 19.0 days, indicating that the low TTR group had a significantly shorter MST and a poorer prognosis (Figure 4).

**Figure 5:**
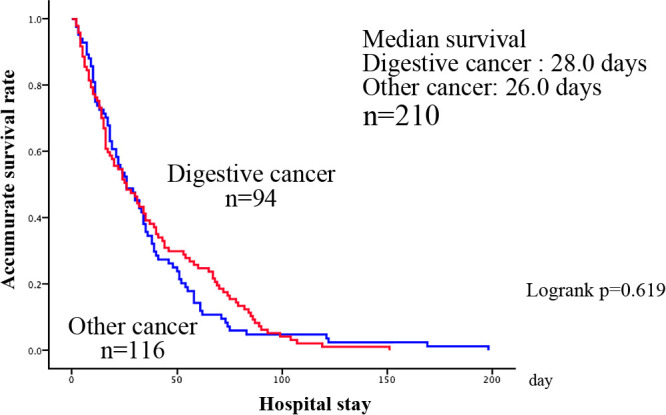
Comparison of Survival curves between gastrointestinal cancer and the other cancer types.

Next, in a comparison between gastrointestinal and non-gastrointestinal cancers, the median (25%–75%) age for the gastrointestinal cancer patients was 78.0 (66–83) years, whereas the mean age for the non-gastrointestinal cancer patients was 76.9±10.9, with a median (25%–75%) age of 77.5 (72–85) years. There were 52 and 42 men and women, respectively, with gastrointestinal cancers and 58 males and women, each, with non-gastrointestinal cancers, showing no age and gender-based differences. Distant metastasis occurred in 64 cases (68.1%) of gastrointestinal cancer and 72 cases (62.1%) of non-gastrointestinal cancer, and recurrence occurred in 66 cases (70.2%) of gastrointestinal cancer and 74 cases (63.8%) of non-gastrointestinal cancer, showing no difference between the two groups. Tumor marker elevations were significantly more frequent in patients with gastrointestinal cancer (68 cases, 72.3%) than in patients with non-gastrointestinal cancer (63 cases, 54.3%) (p=0.007). In gastrointestinal cancer, the median Alb value (25%–75%) on admission was 2.6 (2.2–3.3) g/dL, and the median TTR value (25%–75%) was 11.9 (7.9–13.4) mg/dL, whereas the median Alb value was 2.8 (2.2–3.3) g/dL and the median TTR value (25%–75%) was 12.0 (8.1–18.5) mg/dL in non-gastrointestinal cancer, showing significantly lower values in patient with gastrointestinal cancer (p=0.015, 0.002) (Table 3). The mean survival time (number of days in the hospital) was 41.4±75.1 and 33.4±31.2 days in the patients with gastrointestinal cancer and non-gastrointestinal cancer, respectively (p=0.322) (Table 3). The survival curves showed a MST of 28 and 26 days in the patients with gastrointestinal and non-gastrointestinal cancer, respectively (p=0.619) (Figure 5).

**Figure 6:**
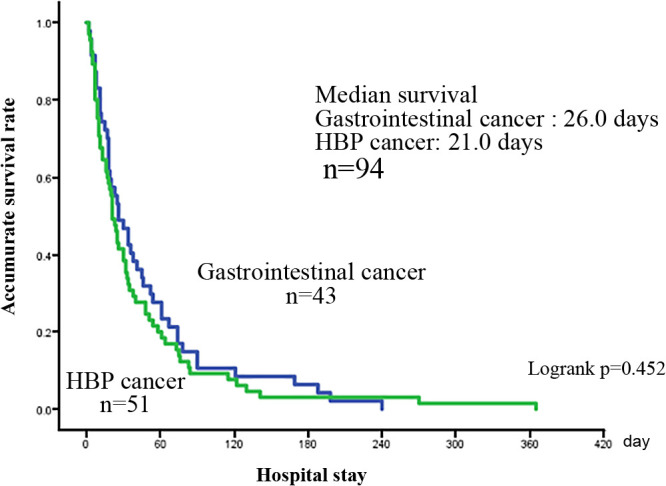
Comparison of Survival curves between hepato-biliary-pancreas (HBP) and gastrointestinal cancer.

Of the 94 patients with gastrointestinal cancers, 51 had hepato-biliary-pancreas cancer and 43 had gastrointestinal tract cancer. There was no significant difference in age between the patients with hepato-biliary-pancreas cancer (median age (25%–75%) 75.0 (67–81) years) and gastrointestinal tract cancer (median age (25%–75%) 76.5 (65–83) years) (p=0.901). There were no significant differences in gender (p=0.701), distant metastasis (p=0.902), cancer recurrence (p=0.313), and tumor marker elevation (p=0.380) between the two groups (Table 4). The median Alb value (25%–75%) was 2.7 (2.2–2.8) g/dL in the patients with hepato-biliary-pancreas cancer and 2.5 (2.3–2.9) g/dL in the patients with gastrointestinal tract cancer, with a significant difference being observed in the lower range for gastrointestinal cancer cases (p=0.049). The median TTR value (25%–75%) was 10.2 (7.3–14.8) mg/dL in the patients with hepato-biliary-pancreas cancer and 8.8 (6.1–13.2) mg/dL in the patients with gastrointestinal tract cancer, showing no significant difference (p=0.268) (Table 4). The mean survival time (number of in the hospital days) (p=0.288) (Table 4) and the MST of the survival curves (p=0.452) (Figure 6) were not significantly different. There were no significant differences in survival time at the median Alb and TTR values between the patients with non-gastrointestinal (p=0.978, 0.938), gastrointestinal tract (p=0.218, 0.062), and hepato-biliary-pancreas cancer (p=0.739, 0.331).

## Discussion

Terminal cancer patients have difficulty in oral ingestion and experience malnutrition because of problems, such as functional disability and cancer cachexia associated with the progression of cancer.^2^ The nutritional status in these patients varies depending on the type of cancer, and patients with gastrointestinal cancers, including stomach, colon, pancreatic, and liver cancer, are thought to often suffer from malnutrition resulting from accompanying symptoms. Moreover, gynecological cancers, such as gastrointestinal and ovarian cancer, often involve peritoneal dissemination, which can cause symptoms like anorexia, nausea and vomiting, and abdominal distention in addition to abdominal pain. In palliative care, nutritional management of patients with terminal cancer with gastrointestinal symptoms is extremely important to avoid further shortening of their remaining prognosis. Thus, it is of utmost importance to prevent the progression of malnutrition. Accordingly, the basic principle of nutritional management for patients with cancer is to provide sufficient energy, administer protein and amino acids to prevent sarcopenia, carry out rehabilitation, and supplement various micronutrients.^3^ Cancer cachexia requires state-specific nutritional management, and it is crucial to accurately grasp the nutritional status and implement a proper management plan. However, while there have been nutritional assessments for various types of cancer, such as gastric, lung, and ovarian cancer,^4–6^ there have been few reports on nutritional assessment based on cancer type. In particular, there have been few reports on the relationship between TTR values and the prognosis of various types of cancer.^7^ Weight loss in patients with cancer is thought to be attributed not only to anorexia but also to increased energy consumption.^8^ Specifically, the following factors are thought to contribute to the mechanism of increased resting energy consumption. First, inflammatory cytokines, such as TNFα and interleukin, and tumor-derived substances, such as lipid-mobilizing factor, may enhance thermogenesis by increasing the expression of uncoupling protein (UCP).^9^

Second, is the activation of the Coli cycle, which is involved in lactate recycling. Tumor cells consume large amounts of glucose through anaerobic metabolism even in anoxic conditions, resulting in the release of large amounts of lactate into the host’s blood. The host converts the lactate into glucose in the liver via the Coli cycle, and this metabolism consumes a large amount of energy (equivalent to 300 kcal per day).^10^ This results in the body losing weight as skeletal muscle protein and adipose tissue are broken down to provide energy in a compensatory manner. Conversely, the onset of cachexia causes skeletal muscle mass to decrease and the synthesis of acute-phase proteins in the liver to increase. However, a decline in albumin synthesis results in hypoalbuminemia. The decrease in skeletal muscle mass is largely caused by increased protein decomposition rather than decreased protein synthesis, and amino acid supplementation alone is unlikely to restore muscle mass.^11^ Therefore, it is necessary to understand the nutritional status and develop management plans suited to each patient’s condition.

This study included 210 patients with terminal cancer (excluding those with refractory cachexia) who were admitted to our department over the 1-year study period and subsequently died or were discharged. The participants were divided into those with gastrointestinal tract cancers and those with non-gastrointestinal cancers. The patients with gastrointestinal cancers were further divided into those with hepato-biliary-pancreas cancer and those with gastrointestinal tract cancer, and their nutritional status at admission and survival time were examined. In a study of 210 patients, the median Alb value (25%–75%) was 2.6 (2.2–3.0) g/dL, which was lower than the lower normal limit, and the median TTR value (25%–75%) was 11.5 (7.9–16.7) mg/dL, which was also lower than the lower normal limit, indicating that the patients with terminal cancer were malnourished. In this study, data from previous studies showed that the cutoff level for TTR values was 22.8 mg/dL for gastric cancer, an average of 180.12±50.16 (mg/L) for lung cancer, and an average of 14.9±9.7 mg/dL for ovarian cancer, which were higher than the data in this study. The lower normal limit for TTR was also 22 mg/dL, but because there were few patients whose values exceeded this level on admission, this parameter could not be used when comparing the two groups.^4–6^ Additionally, although there were reports that extracted and examined cutoffs through ROC analysis,^10^ ROC analysis could not be performed in this study because of the short prognosis in the study population. Histogram analysis showed a large variability in blood data, with a high proportion of patients exhibiting TTR values <10 mg/dL compared with an average of 11.9 mg/dL. Thus, rather than the mean, the median was used in this study. The TTR values, which reflect the current nutritional status more sensitively than Alb values, revealed that patients with poor nutritional status have a shorter prognosis, corroborating our previous report that nutritional disorders are important in predicting prognosis.^12^ In the univariate and multivariate analyses of factors contributing to survival time, age was found to be an independent factor. Age may have contributed to survival because our department provides symptom palliation, nutritional management, and inpatient treatment for refractory fluid retention even for those who are relatively young and can be expected to live long. Moreover, although not significantly different, the TTR value for all data was p=0.064, suggesting that the most recent nutritional status may contribute to survival time.

Therefore, we examined the relationship between nutritional status and cancer type. Of the 210 patients, 94 had gastrointestinal cancers and 116 had non-gastrointestinal cancers. Of the patients with gastrointestinal cancer, 51 had hepato-biliary-pancreas cancer and 43 had gastrointestinal tract cancer. In the two-group comparison of gastrointestinal and non-gastrointestinal cancers, no significant differences were noted in age, gender, distant metastasis, recurrence, or tumor marker elevation. The Alb and TTR values on admission were significantly lower in patients with gastrointestinal cancers than in those with non-gastrointestinal cancers. In the terminal stage of gastrointestinal cancer, patients are in a state of malnutrition, especially from the time of admission. This is considered attributable to the direct effects of cancer, such as reduced food intake and obstruction to food passage, as well as a decline in gastrointestinal functions, such as digestion and absorption. Although no significant differences were observed in survival time or early mortality, the average length of hospital stay was longer for patients with gastrointestinal cancer than for those with non-gastrointestinal cancer, suggesting the importance of understanding, evaluating, and managing nutritional status after the admission of such patients. No significant differences were observed between the patients with hepato-biliary-pancreas cancer and gastrointestinal tract cancer, in age, gender, distant metastasis, recurrence, or tumor marker elevation. The Alb levels on admission were significantly lower in the patients with gastrointestinal tract cancer than in those with hepato-biliary-pancreas cancer, indicating the importance of understanding, evaluating, and managing nutritional state for patients with gastrointestinal tract cancer, especially patients with terminal-stage gastrointestinal cancer, who tend to suffer from malnutrition from the time of admission. Compared with the patients with hepato-biliary-pancreas cancer, the patients with gastrointestinal tract cancer exhibited significantly lower Alb values. Despite an expected high incidence of pancreatic exocrine insufficiency, malnutrition was still present, which might have been directly derived from gastrointestinal tract dysfunction, such as obstruction to passage or nausea. We have reported that a high rate of fat malabsorption is observed after pancreaticoduodenectomy in pancreatic cancer surgery,^13^ and 20%–30% of patients develop fatty liver.^14^ In the present study, the high proportion of pancreatic cancer in the hepato-biliary-pancreas cancer population suggested that attention should be paid to absorption disorders. The pancreatic exocrine function diagnostic test^15^ has been unavailable since March 2021, making it impossible to evaluate pancreatic exocrine function. In our department, pancreatic enzymes are administered orally after surgery for pancreatic cancer, but the evaluation of absorption impairment is a subject for future research. Since no significant differences were observed in early mortality or survival time even in patients with gastrointestinal cancer who were malnourished on admission, it is considered necessary to continue nutritional management without plans of termination.

We also provide nutritional management for patients with terminal cancer, except for those diagnosed with refractory cachexia. Specifically, nutritional screening and assessment are performed on admission, and after nutritional management planning, nutritional support is implemented for all patients. After monitoring and replanning, a reevaluation is performed to provide further nutritional support. Moreover, when refractory cachexia becomes clinically evident, the administration of fluids and energy is restricted to control the burden on the few remaining bodily functions. Since it is practically difficult to detect refractory cachexia on admission, a so-called “time-limited trial” (nutritional management for approximately 1 week, assessment of improvement and deterioration of general condition and nutritional status, and making of the final decision on refractory cachexia) is conducted. Regarding the route of nutrition administration, based on the fundamental principles of nutritional management, oral and enteral nutrition are recommended whenever possible, with intravenous nutrition being used as a supplementary measure. When enteral nutrition cannot be implemented due to factors, such as gastrointestinal tract obstruction, we opt to use a peripherally inserted central venous catheter in combination with intravenous nutrition, which has recently been recommended from the perspective of infection control.^16,17^

In addition to acute conditions observed during surgery or anticancer treatment, malnutrition due to chronic carrier-bearing conditions not only reduces subsequent ADL and QOL but also affects prognosis.^18^ It may be necessary to design tailor-made treatment that also focuses on the type of cancer and dietary intake by carefully evaluating the dietary intake and nutritional state of each patient. Overall, this study has demonstrated that nutritional status is involved in prognosis, as our department has previously proposed, and found that nutritional status differs depending on the type of cancer. Even if a patient was malnourished on admission, there was no significant difference in survival time based on subsequent nutritional management regardless of cancer type. Given that malnutrition is often caused by digestive symptoms, particularly in gastrointestinal cancers, the results suggested that the use of total parenteral nutrition in combination with dietary therapy tailored to each patient’s individual digestive and absorptive status may contribute to improving prognosis.

## Conclusion

The prognosis of patients with terminal cancer with poor nutritional status is unfavorable. Among patients with gastrointestinal cancer, patients with gastrointestinal tract cancer exhibit particularly poor nutritional status, and thus, it is important to provide nutritional management combined with intravenous nutrition according to the individual’s digestive and absorptive condition.

## Figures and Tables

**Table 1:** Background of all patients with terminal cancer

Demographics	total
Disease	
Hepatogastrointestinal cancer	94
hepatocellular carcinoma	6
biliary tract	11
pancreas	34
esophagus	4
gastric	15
colorectal	24
Not hepatogastrointestinal cancer	116
head and neck	13
lung	49
breast	5
gynecological	14
urological	16
other cancers	19
Gender (male/female)	110/ 100
Age median (25–75%)	78.0 (69–84)
Distant metastasis (%)	136/210 (64.8)
Recurrence (%)	140/210 (66.7)
Elevation of tumor marker (%)	131/210 (62.4)
Alb (g/dl) median (25–75%) n=208	2.6 (2.2–3.0)
TTR (mg/dL) median (25–75%) n=200	11.5 (7.9–16.7)
Hospital stay (day) median (25–75%)	24.0 (11.0–48.5)

Albumin: Alb Transthyretin: TTR

**Table 2:** Comparison between gastrointestinal cancer and the other cancer types

n=210	Univariate			Multivariate		
Variables	HR	95% CI	P-value	HR	95% CI	P-value
Age	0.988	0.977–1.000	0.054	0.987	0.975–0.999	0.039
Gender (male/female)	1.062	0.927–1.218	0.385			
Diagnosis (HBP/digestive/other)	0.983	0.667–1.448	0.931			
Distant metastasis	0.989	0.739–1.325	0.943			
Recurrence	0.940	0.703–1.257	0.675			
Tumor marker elevation	1.033	0.693–1.538	0.875			
Alb (g/dl)	0.839	0.674–1.044	0.116			
TTR (mg/dl)	0.979	0.957–1.001	0.064	0.980	0.959–1.002	0.080

Albumin: Alb Transthyretin: TTR

**Table 3:** Comparison between hepato-biliary-pancreas and gastrointestinal cancer

Background on admission	Digestive cancer (n=94)	The other cancer (n=116)	P-value
Gender (male/female)	52/42	58/58	0.443
Age (years) median (25–75%)	78.0 (66–83)	77.5 (72–85)	0.071
Distant metastasis (%)	64/94 (68.1)	72/116 (62.1)	0.510
Recurrence (%)	66/94 (70.2)	74/116 (63.8)	0.380
Elevation of tumor marker (%)	68/94 (72.3)	63/116 (54.3)	0.007
Alb (g/dl) median (25–75%)	2.6 (2.2–3.3)	2.8 (2.2–3.3)	0.015*
TTR (mg/dl) median (25–75%)	11.9 (7.9–13.4)	12.0 (8.1–18.5)	0.002*
Death within 2 weeks	33/94	47/116	0.709
Hospital stay (days) (mean)	41.4±75.1	33.4±31.2	0.322

Albumin: Alb Transthyretin: TTR *: Kruskal Wallis test

**Table 4:** Comparison between hepato-biliary-pancreas (HBP) cancer and gastrointestinal cancer

Background on admission	HBP cancer (n=51)	Gastrointestinal cancer (n=43)	P-value
Gender (male/female)	24/27	19/24	0.701
Age (years) median (25–75%)	75.0 (67–81)	76.5 (65–83)	0.901
Distant metastasis (%)	35/51 (68.6)	29/43 (67.4)	0.902
Recurrence (%)	34/51 (66.7)	32/43 (74.4)	0.313
Elevation of tumor marker (%)	35/51 (68.6)	33/43 (76.7)	0.380
Albumin (g/dl) median (25–75%)	2.7 (2.2–2.8)	2.5 (2.3–2.9)	0.049*
TTR (mg/dl) median (25–75%)	10.2 (7.3–14.8)	8.8 (6.1–13.2)	0.268*
Death within 2 weeks	19/51	14/43	0.208
Hospiral stay (days) (mean)	41.7±59.8	47.9±54.3	0.288

HBP: hepato-biliary-pancreas TTR: Transthyretin *: Kruskal Wallis test

## Supplementary Material

PDF-Japanese
