# Supplementary material for: Investigating the nutritional status characteristics of terminal cancer patients by the type of cancer
Source: Fujita Med J. 2025 Apr 17;11(3):105–10. doi: 10.20407/fmj.2024-007 (PMC12327208; doi:10.20407/fmj.2024-007)
Supplement: Supplementary file 1 — PDF-Japanese [file fmj-11-105-s001.pdf]

Original Article

タイトル：終末期がん患者のがん種別栄養状態の特徴についての検討

ランニングタイトル　終末期がん患者のがん種別栄養状態の検討

藤田医科大学医学部外科・緩和医療学講座
都築則正、臼井正信、二村昭彦、村井美代、伊藤彰博

Norimasa Tsuzuki, MD, PhD*, Usui Masanobu, MD, PhD*, Futamura Akihiko , PhD, Miyo Murai, MD, PhD, Ito Akihiro, MD, PhD

* These two authors contributed equally to this article

Department of Surgery and Palliative Medicine, Fujita Health University, School of Medicine, Toyoake, Aichi, Japan

Original Article

Corresponding author: Usui Masanobu, MD, PhD
Department of Surgery and Palliative Medicine, Fujita Health University,
School of Medicine, 1-98 Dengakugakubo, Kutsukakecho, Toyoake, Aichi
470-1192, Japan
Tel: 0562-93-2111
E-mail: masanobu.usui@fujita-hu.ac.jp

Abstract
目的:終末期がん患者は、がんの進行に伴う悪液質などの問題から低栄養を呈する。特に消化器がんでは消化器症状により低栄養を呈していることが多いが、栄養状態をがん種別に評価し予後まで見通した報告はほとんど無い。そこで今回、入院時のがん種別の栄養評価とその予後について検討した。

方法:2019 年 1 月から 12 月までの 1 年間に、藤田医科大学七栗記念病院緩和ケア病棟に入院し死亡退院した 234 名中入院時に不応性悪液質と判断した 24 名を除く 210 名を対象とし、消化器がん 94 例と非消化器がん 116 例の 2 群に分類し、年齢、性別、転移の有無、初発・再発、入院時の血清 albumin(Alb)値・transthyretin(TTR)値および生存期間について検討した。また、消化器がん 94 例を肝胆膵がん 51 名と消化管がん 43 名に分類して検討を行った。

結果:Alb 値と TTR 値は、消化器がんが非消化器がんに対し有意の低値(p=0.015, 0.002)で、肝胆膵がんと消化管がんでも Alb 値は、肝胆膵がんに対し、消化管がんで有意の低値を示した(p=0.049)。

結語:栄養状態が悪い終末期がん患者は予後不良であり、消化器がんの中でも消化管がんにおいて栄養状態が悪く、個々の消化吸収状態に合わせて静脈栄養を併用した栄養管理を行うことが重要と考えられた。

Key words: 悪液質、栄養状態、終末期がん、消化器がん、消化管がん

【はじめに】

終末期がん患者は、がんの進行に伴う機能的な障害やがん悪液質など様々な問題から経口摂取が困難になり低栄養を呈する。終末期がん患者の多くは、栄養障害を伴う体重減少を認め、すでに悪液質状態に陥っていることが多い。悪液質は、がんをはじめ、慢性心不全や慢性呼吸不全、膠原病など、さまざまな慢性消耗性疾患患者が陥る炎症を背景とした骨格筋量の減少を主徴とする代謝異常である [1)]。特に、がん進展に伴う代謝異常が高度となると、一度減少してしまった体重や筋肉量の回復は困難になる。このため、栄養不良の進行を未然に防ぐことが極めて重要である。すなわち、がん悪液質に対しては、その段階に応じた栄養管理を行うことが必要であり、そのためには、がん患者の栄養状態を的確に把握し適切な計画を立て、実践することが極めて重要である。しかし、そのがん種によって栄養状態に差があることは認知されているが、論文としての報告はほとんどない。特に消化器がんでは付随した特有の消化器症状により低栄養を呈していることが多く、栄養状態をがん種別に評価し予後まで見通した報告は少ない。そこで今回、入院時のがん種別の栄養評価とその予後について検討した。

【方法】

2019 年 1 月から 12 月までの 1 年間に、藤田医科大学七栗記念病院緩和ケア病棟に入院し、入院時に不応性悪液質と判断した 24 名を除く死亡退院した 210 名を対象とした。消化器がん 94 例と非消化器がん 116 例の 2 群に分類し、年齢、性別、転移の有無、初発・再発、入院時の Alb 値・TTR 値および生存期間について検討した。また、消化器がん 94 例を肝胆膵がん 51 名と消化管がん 43 名に分類し、同様の検討を行った。Alb 値については 2 例、TTR 値については 10 例が入院時に測定できていなかった。各群の症例数のアルゴリズムを Figure 1 に示す。2 群間比較は平均値比較を Mann-Whitney の U 検定、中央値比較を Kruskal Wallis 検定を用いて、解析には SPSS.Statistics27(日立 IBM 社)を用いた。

【結果】

入院時に多職種により不応性悪液質かどうかの判断を行い、不応性悪液質と診断され入院後数日(1~6 日)に亡くなった 24 名を除外した 210 名で検討を行った。210 名中、消化器がんは 94 例（食道4、胃 15、大腸 24、膵 34、肝 6、胆道 11 例）で非消化器がんは 116 例（頭頸部 13、肺 49、乳腺5、子宮・卵巣 14、泌尿器 16、その他 19 例）であった(Table 1)。210 名の性別は男性 110 名、女性 100 名であった。年齢の中央値 median(25-75%)は 78.0（69-84）歳であった。遠隔転移は 136 例（64.8%）、再発は 140 例（66.7%）、腫瘍マーカーの上昇は 131 例（62.4%）に認めた。入院時の Alb 値の中央値 median(25-75%)は 2.6 (2.2-3.0) g/dL で、TTR 値の中央値 median(25-75%)は 11.5 (7.9-16.7) mg/dL といずれも正常下限より低値であった。生存期間の中央値 median(25-75%)は 24.0 (11.0-48.5)日で半数以上が 1 ヶ月以内に死亡していた（Table 1）。生存に起因する因子につ

いて単変量・多変量解析を行うと、単変量解析では年齢とTTR 値が生存に寄与する傾向を認め、多変量解析では年齢のみが独立した因子であった（Table 2）。TTR 値は、多変量解析においても生存に寄与する傾向を認めた。TTR 値と生存期間の関連性に着目し、栄養評価として Alb 値とTTR 値の解析を行った。終末期がん患者では血液データにばらつきが多くヒストグラムを示す(Figure 2)。TTR 値は平均 11.9 mg/dL であるが 10 mg/dL 以下の割合が高かった。入院時のAlb 値とTTR 値はばらつきが多いため、中央値で検討を行った。全体208例のAlb 値の中央値は2.6 g/dL、200例の TTR 値の中央値は 11.5 mg/dL であった。生存曲線では、Alb 値が中央値2.6 g/dL より高い群(Alb 高値群)で median survival time (MST)25.0 日に対し、2.6 g/dL 以下の群は MST 20.0 日と Alb 高値群が長い傾向を示したが、2 群間に有意差は認めなかった(Figure 3)。これに対し、TTR 値は、中央値 11.5 mg/dL より高い群(TTR 高値群)が MST 26.0日に対し、11.5 mg/dL 以下の群(TTR 低値群)はMST 19.0 日と、TTR 低値群で有意に短く、予後不良であった(Figure 4)。

次に消化器がんと非消化器がんの比較では、年齢は消化器がんで中央値 median(25-75%) 78.0（66-83）歳で、非消化器がんは平均76.9±10.9で中央値median(25-75%) 77.5（72-85）歳であった。性別は消化器がんは男性 52 名、女性 42 名で非消化器がんが男性 58 名、女性 58 名と年齢・性別に差は無かった。遠隔転移は、消化器がん64例（68.1%）、非消化器がん72例（62.1%）、また再発は消化器がん66例（70.2%）、非消化器がん74例（63.8%）といずれも2群間に差は認めなかった。腫瘍マーカーの上昇は消化器がん 68 例（72.3%）に対し、非消化器がん 63 例(54.3%)と消化器がんで有意に多かった(p=0.007)。入院時の Alb 値の中央値 median(25-75%)は、消化器がん 2.6 (2.2-3.3) g/dL で、TTR 値の中央値 median(25-75%)は、11.9 (7.9-13.4) mg/dL に対し、非消化器がんで Alb 値の中央値 2.8 (2.2-3.3) g/dL、TTR 値の中央値 median(25-75%) 12.0 (8.1-18.5) mg/dL と消化器がんで有意の低値を示した(p=0.015, 0.002)(Table 3)。生存期間（在院日数）の平均は消化器がんで 41.4±75.1 日に対し、非消化器がんで 33.4±31.2 日であった(p=0.322) (Table 3)。生存曲線では、生存期間の中央値が消化器がんで 28 日、非消化器がんで26 日であった(p=0.619) (Figure 5)。

消化器がん94名のうち、肝胆膵がんは51名で消化管がんは43名であった。年齢は肝胆膵がんが中央値median(25-75%) 75.0（67-81）歳で、消化管がんが中央値median(25-75%) 76.5（65-83）歳と有意差を認めなかった(p=0.901)。性別(p=0.701)、遠隔転移(p=0.902)、再発(p=0.313)、腫瘍マーカーの上昇(p=0.380)は 2 群間に有意差は認めなかった(Table4)。Alb 値の中央値median(25-75%)は、肝胆膵がんが 2.7 (2.2-2.8) g/dL、消化管がんが 2.5 (2.3-2.9) g/dL と消化管がんが低値で有意差を認めた(p=0.049)。TTR 値の中央値 median (25-75%)は、肝胆膵がんが 10.2 (7.3-14.8) mg/dL、消化管がんが 8.8 (6.1-13.2) mg/dL で有意差を認めなかった(p=0.268) (Table

4)。生存期間（在院日数）の平均(p=0.288) (Table 4)と、生存曲線の生存期間中央値(p=0.452) (Figure 6)は、有意差を認めなかった。Alb 値と TTR 値の中央値における生存期間においても、非消化器がん(p=0.978, 0.938)、消化管がん(p=0.218, 0.062)、肝胆膵がん(p=0.739, 0.331)で有意差を認めなかった。

【考察】

終末期がん患者は、がんの進行に伴う機能的な障害やがん悪液質など様々な問題から経口摂取が困難になり低栄養を呈する[2]。がん種によっては栄養状態に差があり、胃癌、大腸癌、膵癌、肝癌をはじめとする消化器がんは付随する症状により低栄養を認めることが多いと考えられる。さらに消化器がんや卵巣癌などの婦人科領域がんは腹膜播腫を認めることも多く、腹痛以外にも、食欲不振、嘔気・嘔吐、腹部膨満感などの症状も認める。緩和医療において消化器症状を認める終末期がん患者の栄養管理は極めて重要で、残された予後を更に短くしないことが求められる。このため、栄養不良の進行を未然に防ぐことが極めて重要であり、がん患者に対する栄養管理の原則は、十分量のエネルギー補給に加えてサルコペニア予防を目的とした蛋白・アミノ酸の投与と、リハビリテーションの併施や各種微量栄養素の補充である[3]。がん悪液質に対して、状態に応じた栄養管理が必要であり、栄養状態の的確な把握と適切な管理計画による実践が重要となる。しかし、これまで胃がん、肺がん、卵巣がんなどのがん種別での栄養評価はあるが[4–6]、がん種別の栄養評価を行った報告はほとんどなく、特に栄養状態のなかでも TTR 値とがん種別の予後に関する報告はほとんどない[7]。がん患者の体重減少の要因は食欲不振のみならずエネルギー消費の亢進にあると考えられている[8]。すなわち、安静時エネルギー消費が亢進する機序として以下のような複数の要因が考えられている。第一に、TNF α や Interleukin などの炎症性サイトカインや LMF（lipid-mobilizing factor）のような腫瘍由来物質が uncoupling protein (UCP)の発現の増強による熱産生亢進の可能性がある[9]。

第二に、乳酸のリサイクリングに関与する Coli サイクルの活性化が挙げられる。腫瘍細胞は、無酸素状態においても、嫌気的代謝によって大量のグルコースを消費するため、宿主の血中に多くの乳酸が放出される。宿主はその乳酸を肝臓で Coli サイクルによってグルコースに変換するが、この代謝には大量のエネルギー（1 日 300kcal 相当）を消費する[10]。このため，代償的に骨格筋蛋白や脂肪組織を分解してエネルギーを賄うため、体重が減少していく。一方、悪液質の発現により骨格筋は減少し、肝臓の急性期蛋白の合成は増加するが、アルブミン合成は低下するため低アルブミン血症を認める。骨格筋の減少は、蛋白合成の低下より分解の増加による要因が大きく、アミノ酸の補給のみで筋量の回復は見込めない[11]。このため、状態にあった栄養把握と管理計画が必要となる。

今回 1 年間に当科に入院後死亡退院した、不応性悪液質を除く終末期がん患者 210 名を対象とし、消化器がんと非消化器がん、消化器がんをさらに肝胆膵がんと消化管がんに分類し、入院時の栄養状態と生存期間について検討した。210 名の検討では、Alb 値は中央値(25-75%)は 2.6 (2.2-3.0) g/dL と正常下限値より低く、TTR 値も中央値(25-75%)は 11.5 (7.9-16.7) mg/dL とこれも正常下限値より低く終末期がん患者は低栄養であった。今回の検討に当たり、これまでの論文のデータでは、TTR 値のデータは、胃がんでカットオフ値 22.8mg/dL、肺がんで平均 180.12±50.16 (mg/L)、卵巣がんで平均 14.9±9.7 mg/dL と今回のデータに比べて高く、正常下限値も TTR が 22mg/dL であるが、入院時にこのデータを上回る患者はほとんどいないため、これも2群比較には使用できなかった[4-6]。また、ROC 解析による cut off を抽出し検討した報告も認めるが[10)]、今回の検討では予後が短く ROC 解析ができなかった。ヒストグラム解析では血液データのばらつきが大きく、TTR 値は平均 11.9mg/dL に対し, 10mg/dL 以下の割合が多かった。このため今回は平均ではなく中央値で検討を行った。Alb よりもさらに鋭敏に現在の栄養状態を反映する TTR 値でみると、栄養状態が悪い患者で予後が短いことが確認され、これまで栄養障害が予後予測に重要であるとしてきたわれわれの報告が裏付けられた[12)]。生存期間に寄与する因子の単変量・多変量で解析では、年齢が独立した因子であった。当科では、比較的若く、死亡するまでの期間が期待できる方も症状緩和や栄養管理、難治性体液貯留に対する入院治療を行うことから、年齢が生存期間に寄与したと考えられた。また、有意差は認めなかったが、全データの TTR 値は p=0.064 であり、直近の栄養状態が生存期間に寄与する可能性が示唆された。

そこで、栄養状態とがん種別の関係について検討した。210 名中、消化器がんは 94 例、非消化器がんは 116 例であった。消化器がんは、肝胆膵がんが 51 例、消化管がんが 43 例であった。消化器がんと非消化器がんの2群比較では、年齢、性別、遠隔転移、再発、腫瘍マーカー上昇に有意差は認めなかった。入院時の Alb 値と TTR 値は、消化器がんで非消化器がんに比べ有意に低値であった。消化器がんの終末期は特に入院時より低栄養状態であり、がんによる食事摂取量の低下や通過障害などの直接的な影響と、消化吸収などの消化器機能の低下が要因として考えられる。一方、生存期間や早期死亡率に有意差は認めなかったが、平均在院日数は非消化器がんに比べ、消化器がんの方が長く、入院後の栄養状態の把握と評価管理が重要であると考えられた。肝胆膵がんと消化管がんの検討でも、年齢、性別、遠隔転移、再発、腫瘍マーカー上昇に有意差は認めなかった。入院時の Alb 値は、消化管がんが肝胆膵がんに比べ有意に低値で、消化器がんの中でも、特に消化管がんの終末期は入院時より低栄養状態で、栄養把握と評価管理が重要であることが示された。肝胆膵がんと消化管がんの比較において、消化管がんは Alb 値が有意に低値で、膵外分泌機能不全が多いことが予想されるにもかかわらず低栄養なのは、直接的な通過障害や嘔気などの消化管機能不全が原因である可能性が推察される。膵癌手術の膵

頭十二指腸切除の術後には高率に脂肪吸収不全を認め[13)], 20%～ 30%が脂肪肝になることを報告してきた[14)]。今回の検討では、肝胆膵がんに占める膵癌の割合が高いため、吸収障害に注意が必要であると考えられた。膵臓の外分泌検査の PFD 試験(pancreatic function diagnostic test)[15)] が 2021 年3月より使用できず、膵外分泌機能が評価できなくなった。当科では、膵癌の手術後に膵酵素を内服しているが、吸収障害の評価については今後の検討課題である。入院時低栄養である消化器がんにおいても早期死亡や生存期間に有意差を認めなかったため、あきらめずに栄養管理を行う必要があると考えられた。

われわれは、終末期がん患者に対しても不応性悪液質(refractory cachexia)と診断した患者以外は栄養管理を行っている。具体的には、入院時に栄養スクリーニング・アセスメントを行い、栄養管理プランニング後に、栄養管理を全例に実施し、モニタリングや再プランニング後に再評価を行い、栄養サポートを進めている。また, 悪液質の中でも refractory cachexia が臨床的に明確になった場合、水分やエネルギーの投与を抑制し、残されたわずかな身体機能への負荷を制御している。入院時に不応性悪液質を見抜くのは現実的には困難であり、いわゆる“time limited trial” (1 週間程度の栄養管理、全身状態、栄養状態の改善と悪化を見極め refractory cachexia の最終判断)を施行している。栄養投与ルートに関しても、栄養管理の大原則に基づき、できる限り経口・経腸栄養を推奨し、静脈栄養は補助的手段として行い、消化管の通過障害などで、経腸栄養が実施できない場合、最近では感染管理の観点からも推奨されている末梢挿入型中心静脈カテーテルを留置して静脈栄養の併用を選択している[16,17)]。

手術や抗がん剤治療中のような急性期の病態だけでなく、慢性的な担がん状態による低栄養はその後の ADL や QOL の低下のみならず、予後にも影響する[18)]。食事摂取量や栄養評価を個々の患者について注意深く評価を行って、がん種や食事摂取にも着目したテーラーメード治療が必要であることが考えられる。今回の検討で、全体では、当講座がこれまで提唱してきたように、予後に栄養状態が関与していることが示され、それぞれのがん種によって栄養状態がそれぞれ別々であることがわかった。入院時に低栄養でもその後の栄養管理でがん種による生存期間に有意差はなく、特に消化器がんでは消化器症状による低栄養が多いため、中心静脈栄養を併用し、個々の消化吸収状態に合わせた食事療法を行うことで、予後の改善に寄与する可能性が示唆された。

【結語】

栄養状態が悪い終末期がん患者は予後不良であり、消化器がんの中でも消化管がんにおいて栄養状態が悪く、個々の消化吸収状態に合わせて静脈栄養を併用した栄養管理を行うことが重要と考えられた。

【利益相反】

この論文作成に当たり、利益相反はありません。

参考文献

1) Fearon K, Strasser F, Anker SD, et al. Definition and classification of cancer cachexia: an international consensus. Lancet Oncol 2011;12:489-95.
2) Baracos VE, Martin L, Korc M, Guttridge DC, Fearon KCH. Cancer-associated cachexia. Nat Rev Dis Primers 2018;4:17105.
3) Arends J, Bachmann P, Baracos V, et al. ESPEN guidelines on nutrition in cancer patients. Clin Nutr 2017;36:11-48.
4) Shimura T, Shibata M, Gonda K, Okayama H, Saito M, Momma T, Ohki S, Kono K. Serum transthyretin level is associated with prognosis of patients with gastric cancer. J Surg Res 2018;227:145-50.
5) Ding H, Liu J, Xue R, Zhao P, Qin Y, Zheng F, Sun X. Transthyretin as a potential biomarker for the differential diagnosis between lung cancer and lung infection. Biomed Rep 2014;2:765-9.
6) Gericke B, Raila J, Sehouli J, Haebel S, Könsgen D, Mustea A, Schweigert FJ. Microheterogeneity of transthyretin in serum and ascitic fluid of ovarian cancer patients. BMC Cancer 2005;5:133.
7) Bozzetti F, Migliavacca S, Scotti A, Bonalumi MG, Scarpa D, Baticci F, Ammatuna M, Pupa A, Terno G, Sequeira C, Masserini C, Emanuelli H. Impact of cancer, type, site, stage and treatment on the nutritional status of patients. Ann Surg 1982;196:170-9.
8) Bianchi A, Bruce J, Cooper AL, Childs C, Kohli M, Morris ID, Morris-Jones P, Rothwell NJ. Increased brown adipose tissue activity in children with malignant disease. Horm Metab Res 1989;21: 640-1.
9) Collins P, Bing C, McCulloch P, Williams G. Muscle UCP-3 mRNA levels are elevated in weight loss associated with gastrointestinal adenocarcinoma in humans. Br J Cancer 2002;86: 612-8.
10) Esper DH,Harb WA. The cancer cachexia syndrome: a review of metabolic and clinical manifestations. Nutr Clin Pract 2005;20: 369-76.
11) Tisdale MJ. Cachexia in cancer patients. Nat Rev Cancer 2002;2: 862-71.
12) Murai M, Higashiguchi T, Futamura A,Ohara H, Tsuzuki N, Itani Y, Kaneko T, Chihara T, Shimpo K, Nakayama N. Interleukin-8 and clinical symptoms can be prognostic indicators for advanced cancer patients with cachexia. Fujita Med J 2020; 6: 117-21.
13) Murata Y, Mizuno S, Kato H, Kishiwada M, Ohsawa I, Hamada T, Usui M, Sakurai H, Tabata M, Nishimura K, Fukutome K, Isaji S. Nonalcoholic steatohepatitis (NASH) after pancreaticoduodenectomy: association of pancreatic exocrine deficiency and infection. Clin J Gastroenterol 2011;4:242-8.
14) Kato H, Isaji S, Azumi Y, Kishiwada M, Hamada T, Mizuno S, Usui M, Sakurai H, Tabata M. Development of nonalcoholic fatty liver disease (NAFLD) and nonalcoholic steatohepatitis

(NASH) after pancreaticoduodenectomy: proposal of a postoperative NAFLD scoring system. J Hepatobiliary Pancreat Sci 2010;17:296-304.

15) Kato K, Isaji S, Kawarada Y, Hibasami H, Nakashima K. Effect of zinc administration on pancreatic regeneration after 80% pancreatectomy. Pancreas 1997;14:158-65.

16) Cederholm T, Barazzoni R, Austin P, et al. ESPEN guidelines on definitions and terminology of clinical nutrition. Clin Nutr 2017;36:49-64.

17) Radbruch L, Elsner F, Trottenberg P, Baracos V, Fearon K. Clinical practice guidelines on cancer cachexia in advanced cancer patients with a focus on refractory cachexia. European Palliative Care Research Collaborative; 2010.

18) F. Bozzetti, J. Arends, K. Lundholm, A. Micklewright, G. Zurcher, M. Muscaritoli. ESPEN Guidelines on Parenteral Nutrition: non-surgical oncology. Clinical Nutr 2009; 28 , 445-54.

Legend for figures

Figure 1 Algorithm of terminal cancer patients for type of cancer in Nanakuri Memorial Hospital from January 2019 to December 2019.

Figure 2 Histogram of serum albumin and transthyretin levels in terminal cancer patients.

Figure 3 Survival curves of terminal cancer patients according to albumin level.

Figure 4 Survival curves of terminal cancer patients according to transthyretin level.

Figure 5 Comparison of Survival curves between digestive cancer and the other cancer.

Figure 6 Comparison of Survival curves between hepato-biliary-pancreas (HBP) cancer and gastrointestinal cancer.

Table 1 Background of all terminal cancer patients.

Table 2 Comparison between digestive cancer and the other cancer.

Table 3 Comparison between hepato-biliary-pancreas cancer and gastrointestinal cancer.

Table 4 Comparison between HBP cancer and gastrointestinal cancer.
